# Immunotherapy: A promising novel endometriosis therapy

**DOI:** 10.3389/fimmu.2023.1128301

**Published:** 2023-04-17

**Authors:** Wenshu Li, Aimin Lin, Lin Qi, Xin Lv, Shenghuan Yan, Jing Xue, Nan Mu

**Affiliations:** ^1^ Departments of Gynecology and Obstetrics, Yantai Yuhuangding Hospital Affiliated to Qingdao University, Yantai, Shandong, China; ^2^ Departments of Gynecology and Obstetrics, Shandong Provincial Hospital affiliated to Shandong University, Jinan, Shandong, China; ^3^ Specialized Laboratory of Gynecology and Obstetrics, Yantai Yuhuangding Hospital Affiliated to Qingdao University, Yantai, Shandong, China

**Keywords:** endometriosis, pathogenesis, therapeutic strategy, inflammatory factor, immunology

## Abstract

Endometriosis is a common disease of the female reproductive system and has malignant features. Although endometriosis by itself is a benign disease, its erosive growth characteristics lead to severe pelvic pain and female infertility. Unfortunately, several aspects of the pathogenesis of endometriosis are still unclear. Furthermore, the clinical therapeutic methods are unsatisfactory. The recurrence rate of endometriosis is high. Accumulating evidence suggests that the onset and development of endometriosis are closely related to the abnormal function of the female autoimmune system, especially the function of some immune cells such as the aggregation of neutrophils, abnormal differentiation of macrophages, decreased cytotoxicity of NK cells, and abnormal function of T- and B-cell lines. Therefore, immunotherapy is probably a novel therapeutic strategy for endometriosis besides surgery and hormone therapy. However, information regarding the clinical application of immunotherapy in the treatment of endometriosis is very limited. This article aimed to review the effects of existing immunomodulators on the development of endometriosis, including immune cell regulators and immune factor regulators. These immunomodulators clinically or experimentally inhibit the pathogenesis and development of endometriosis lesions by acting on the immune cells, immune factors, or immune-related signaling pathways. Thus, immunotherapy is probably a novel and effective clinical treatment choice for endometriosis. Experimental studies of the detailed mechanism of immunotherapy and large-scale clinical studies about the effectiveness and safety of this promising therapeutic method are required in the future.

## 1 Introduction

Endometriosis is one of the most common benign diseases affecting women of reproductive age. It is estimated that 10%–15% of women worldwide suffer from endometriosis (1). Endometriosis is characterized by the development of functional endometrial tissue outside of the endometrium in the uterine cavity and the myometrium of the uterine body (2). Because of the uncertainty of the growth site of endometriosis lesions, the clinical symptoms of endometriosis vary widely. The most common clinical symptoms are dysmenorrhea, chronic pelvic pain, and infertility, which severely affect the patients’ quality of life.

The revised American Society for Reproductive Medicine (rASRM) made clinical staging classification of endometriosis based on the location, number, range, depth, and degree of adhesion of the endometrial lesions. The staging standard widely accepted by clinicians is the rAFS staging standard revised by the rASRM in 1997. Endometriosis grading is divided into four stages: I, minimal; II, mild; III, moderate; and IV, severe (3). This grading system of endometriosis provides a basis for evaluating disease development and selecting clinical treatment strategies. At present, the treatment principle of endometriosis is to regulate the menstrual cycle, relieve pain, restore fertility, and prevent recurrence. According to this principle, surgical treatment and hormone therapy are widely accepted in the treatment of endometriosis. Unfortunately, the etiology of endometriosis is still unclear. Furthermore, there is no clinically satisfactory therapeutic method. Accumulating documents suggest that the immune factors probably play an important role in the pathogenesis of this disease, and immunotherapy might be a promising strategy in the treatment of endometriosis. In the current article, we have reviewed the potential immunotherapy strategies for endometriosis that may provide a novel insight into the treatment of this benign disease with malignant characteristics.

## 2 The role of immunocompetent cells in the pathogenesis of endometriosis

The pathogenesis of endometriosis is a complicated process. Several pathological factors make contributions to the development of this disease. In addition, a genetic associated mechanism also seems to be involved in the pathogenesis of endometriosis. A study on patients with twin endometriosis once pointed out that the risk of this disease caused by genetic variation is higher than that of the normal population, which suggested that the incidence of endometriosis also has genetic characteristics (4). At this stage, the comprehensive analysis of human gene chain will further reveal the gene sites related to the pathogenesis of endometriosis (5). At present, the most widely accepted theory for the etiology of endometriosis is “menstrual countercurrent,” which is proposed by Sampson: during menstruation, the active endometrial cells and tissue fragments enter the pelvic cavity through the contraction of the fallopian tube along with menstrual blood and attach to the surface of the pelvic structure (6). This attachment induces a local immune response and fibrosis. The innate immune cells involved in the immune response process include neutrophils, macrophages, NK cells, and T cells. In the following sections, we will briefly explain the role played by these immune factors in the pathogenesis of endometriosis.

## 3 Neutrophils

The number of neutrophils in the abdominal cavity of patients with endometriosis is higher than that of healthy controls (7). Especially in the early stage when endometrial cells/tissues invade and damage the pelvic tissues, the aggregation of neutrophils reaches a peak, which also conforms to the cytological characteristics of acute inflammatory reaction (8). The aggregation of neutrophils may be due to the increased concentrations of chemokines such as IL-8, epithelial neutrophil activating peptide (ENA-78), and human neutrophil peptide (HNP1-3) in the plasma and peritoneal fluid in the local endometriosis environment (9). The inhibition of excessive neutrophil aggregation in the abdominal cavity was found to be associated with reduction of endometriosis lesion formation in animal experiments (10). Furthermore, the inflammatory reaction caused by neutrophil aggregation in pelvic endometriosis lesions is also reported to be responsible for the pelvic pain in affected patients.

## 4 Macrophages

The number of macrophages in the peritoneal fluid and normal endometrial tissue of patients with endometriosis is higher than that of healthy controls (11). Literature suggests that macrophages are probably involved in the development of endometriosis through the following ways (12):

1. *Inflammatory reaction induction*. In endometriosis lesions, macrophages and mast cells have been found to release chemokines (such as TNF, IL-6, and IL-1 β) that play a key role in the neutrophil recruitment process (11, 13).
2. *Promotion of endometrial cell proliferation*. It was reported that co-culture of endometrial stromal cells and macrophages promotes the proliferation of endometrial stromal cells *in vitro* (14, 15).
3. *Promotion of formation of new blood vessels in endometriosis lesions*. Increased macrophages in the abdominal cavity are believed to be associated with the accumulation of vascular endothelial growth factor in endometriosis lesions, which promotes the growth of new blood vessels in endometriosis lesions (16).
4. *Attenuated phagocytosis*. Many studies have reported that the phagocytosis of macrophages in patients with endometriosis is weakened. The attenuated phagocytosis of peritoneal macrophages isolated from patients with endometriosis was reported to be related to the decreased expression of differentiation cluster 36 (CD36) (17). The decrease of macrophage phagocytosis ultimately weakens the growth inhibition of endometrial cells. In addition, some researchers have pointed out that different types of macrophages (MI type and MII type) played different roles in the development of endometriosis (11, 18).

## 5 NK cells

NK cells are cytotoxic effector lymphocytes in the human body. They are divided into two types: the typical CD56brightCD16− NK cells that are characterized as high-level cytokine producers, and CD56dimCD16+ NK cells that are characterized as highly cytotoxic (19). In endometriosis, the high cytotoxicity of the CD56dimCD16+ NK cells in the peripheral blood and peritoneal fluid was inhibited (20–22). The reduction of cytotoxicity leads to the immune escape of ectopic endometrium fragments in the peritoneal cavity, making it easier for ectopic endometrial cells to survive in the abdominal cavity, and ultimately promoting the pathogenesis of endometriosis. Moreover, the decrease of NK cytotoxicity in patients with endometriosis is suggested to be affected by cytokines (TGF-β, IL-6, and IL-15) in the peritoneal fluid (23–25).

## 6 T cells

T cells are the response cells of human cellular immunity, and the important family member CD4+ T cells are categorized into four subsets: TH1, TH2, TH17, and regulatory T cells (Tregs). TH2, TH17, and Tregs have been reported to be highly expressed in the peritoneal fluid of patients with endometriosis (26–28). Th2-related cytokines (e.g., IL-4 and TSLP) and IL-17a secreted by TH17 cells have been described as aggravators for endometriosis development, given their inflammation-aggravating and endometrial cell proliferation-promoting effects (12, 29). The increase in the quantity of Tregs in the peritoneal fluid of the patients may enhance local immunosuppression and inhibit the elimination of ectopic endometrial cells (30). In addition, Tregs were found to impair infertility and aggravate pelvic pain in endometriosis patients (31).

## 7 Mast cells

Mast cells are important immune cells responding to human allergic reaction. At the initial stage of the inflammatory reaction, the lesions will recruit mast cell precursor cells from the circulatory chemokines to gather around the endometrial issue. Mast cells also play an important role in the pathogenesis of endometriosis. Some experimental data showed that a large number of mast cells infiltrate around endometrial stromal cells in endometriosis tissues, especially around the blood vessels and fibrous stroma (32, 33). It was also found that the number of degranulated mast cells in endometriosis lesions is significantly higher than that in normal endometrial tissue (33), and the important differentiation factor of mast cells has also been reported to show an increased expression in the abdominal cavity of patients with endometriosis (34). Therefore, mast cells are suggested to be a key regulator in the pathogenesis of endometriosis.

## 8 Dendritic cells

Dendritic cells (DCs) are a major type of antigen-presenting cells that are specially used for the initiation and regulation of adaptive immune response. Recent research shows that the density and number of DCs in the endometrium and ectopic endometrium of patients with endometriosis have increased (35). The role and underlying mechanism of DCS in endometriosis are still unclear. Some studies have reported that plasma cell-like DCs enhance endothelial cell migration and promote angiogenesis and pathological growth of endometrial tissue by secreting IL-10 (36). Some clinical studies also pointed out that the proportion of immature DCs in the peritoneum of patients has increased, which reduced the antigen-presenting ability of DCs, causing the immune escape of endometrial cells to the peritoneal cavity, facilitating the spread and growth of endometrial tissue in the abdominal cavity (37).

## 9 Immunotherapy of endometriosis

**Figure 1:**
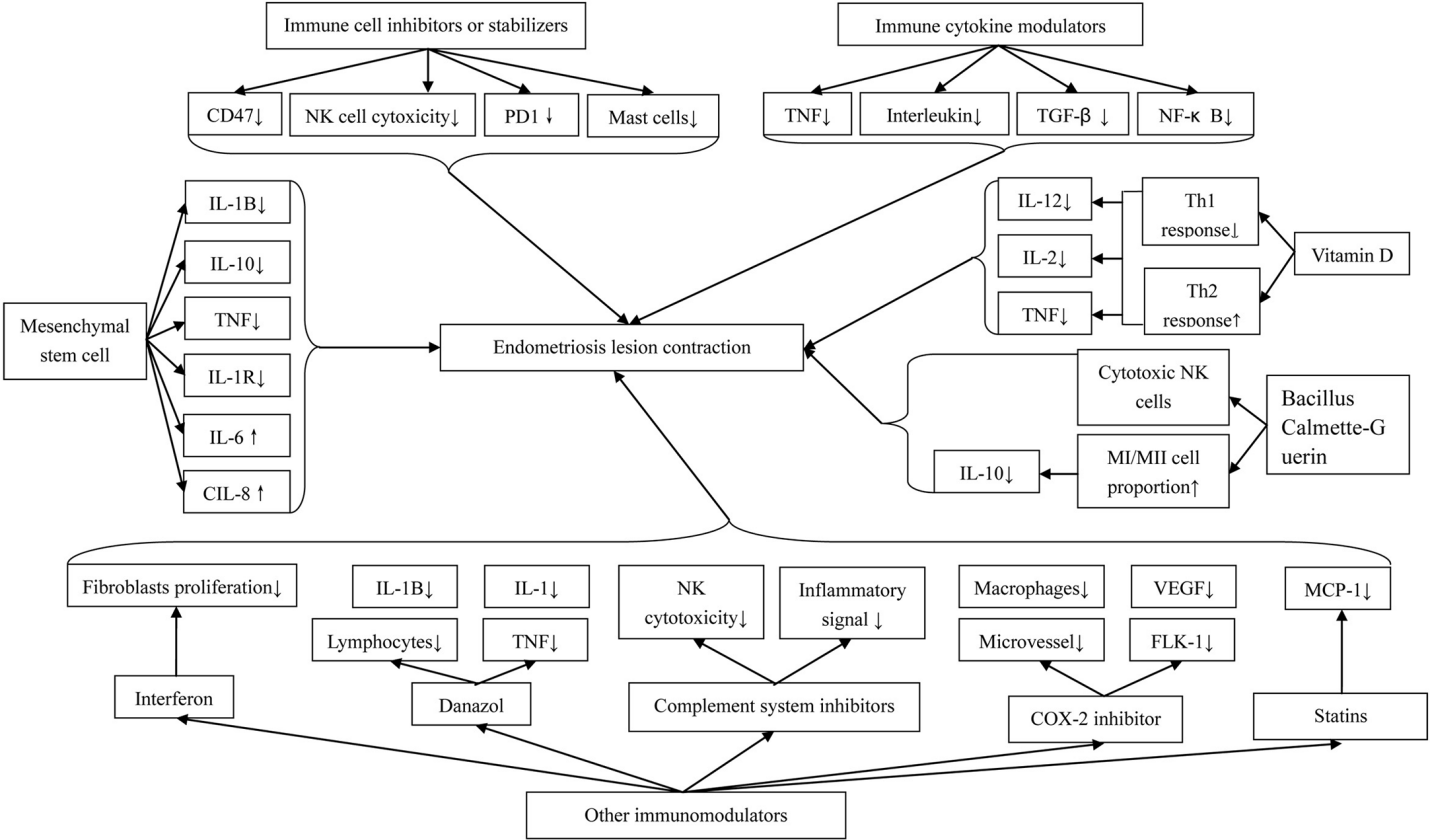
Immunotherapeutic Methods for Endometriosis.

The immune system seems to play an important role in the pathogenesis and development of endometriosis. Hence, immune therapy is probably a promising method for endometriosis treatment (**Figure 1**).

### 9.1 Immune cell inhibitors or stabilizers

The decrease of macrophagic phagocytosis in the serum and peritoneal fluid of patients with endometriosis is an important promoter causing immune imbalance. The expression of CD47 is used by macrophages to distinguish “self” or “non-self” cells. The high expression of CD47 promotes the release of the “don’t eat me” signal of cells, which is a key process in the immune escape (38). The CD47 site inhibitor blocks this signal and allows macrophages to perform normal phagocytosis. It was found clinically that the CD47 level in the ectopic endometrium of patients with endometriosis was significantly increased (39). By using siRNA or neutralizing antibodies to reduce the expression of CD47, phagocytosis of macrophages was found to be improved *in vitro*. Immunotherapy based on the CD47SIRPa signaling pathway seems to be effective in the treatment of endometriosis. In addition, exosomes from endometriosis were reported to promote the phenotype of macrophages into MII polarization, and weaken the phagocytosis of macrophages *in vivo* and *in vitro*. Therefore, anti-exocrine therapy for patients with endometriosis seemed to significantly promote the recruitment of macrophages to ectopic lesions, reduce the proportion of MII-type phagocytes, and ultimately enhance the phagocytosis of macrophages to ectopic endometrial cells (40, 41).

The inhibition of NK cell cytotoxicity is related to the pathogenesis of endometriosis. Activating NK cell cytotoxicity is a possible new therapeutic strategy for endometriosis. In an animal experiment, researchers found that oral probiotics significantly activate abdominal NK cells and reduce the formation of endometriosis (42). An extract of the white mistletoe tree Helixor A also directly activates the cytotoxicity of NK cells and was found to significantly inhibit the development of endometriosis tissue (43).

Endometriosis stimulates bone marrow mesenchymal stem cell differentiation through paracrine signal transduction and increases the expression of PD-1 in T cells. The increased expression of PD-1 may be a mechanism by which the endometrial tissue avoids immune monitoring. Targeted inhibition of PD-1 may be an effective method in the treatment of endometriosis (44).

We mentioned previously that the number of mast cells, especially activated mast cells, in animal and human endometrial tissue was significantly increased. Therefore, the application of stabilizers and inhibitors that inhibit mast cell aggregation and differentiation is probably effective in the treatment of endometriosis. Thus far, existing medical methods targeting MC are divided into two groups: the first group comprises drugs that only act as MC stabilizers, and the second group comprises drugs that have dual effects of MC stabilizer and histamine 1 receptor antagonist (45). The drugs or pathways that enter the clinical trial are palmitoyl ethanolamide and levonorgestrel-releasing intrauterine system (LNG IUS) and are in the animal experiment stage: Zafirlukast, sodium cromoglycate (or sodium cromolyn), and JAK2 inhibitor tyrphostin: JAK/STAT pathway (46).

As an immunomodulator, loratadine regulates the immune cells in various endometrial tissues and peritoneal fluid. This agent stimulates the activity of macrophages and T cells, enhances NK cell cytotoxicity, and stimulates the proliferation of B cells. These activities subsequently resist the body-mediated cytotoxicity. It was reported that IL-1α, TNF, TFN-β, IL-6, interferon-α, and interferon-γ are all likely targets of loratadine clinically (47, 48).

### 9.2 Immune cytokine modulators

#### 9.2.1 Anti-TNF preparation

TNF is the main product of activated macrophages. This inflammatory factor activates inflammatory leukocytes and promotes the production of other proinflammatory cytokines such as IL-1, IL-6, and other TNF. It was reported that this inflammatory factor is involved in the pathogenesis of endometriosis (49, 50). Inhibition of TNF levels seemed to have beneficial effects on endometriosis. Clinically, this treatment effect is usually achieved by decreasing the TNF levels or by blocking the signaling pathway of TNF on target tissues. Currently, anti-TNF-related agents include pentoxifylline, leflunomide, etanercept, infliximab, and recombinant human TNF binding protein-1 (r-hTBP-I). Among them, pentoxifylline is a multisite immunomodulatory drug that affects the production of inflammatory mediators and the responsiveness of immune active cells to inflammatory stimuli. The mechanism of this effect is probably that pentoxifylline inhibits phagocytosis and toxic oxygen production and reduces inflammation of TNF and IL-1 (51). In an animal study, pentoxifylline was found to significantly reduce the growth of endometriosis implants in rats (52). Etanercept is a fusion protein composed of human recombinant soluble TNF receptor-2 (p75) and human Fc antibody subunit. Etanercept can neutralize the activity of TNF and is currently used to alleviate the symptoms of autoimmune diseases clinically (39). Data by Braun et al. showed that etanercept blocks the ability of the peritoneal fluid in endometriosis to enhance the proliferation of eutopic or ectopic endometrial cells (53). In addition, recombinant human TNF binding protein I (r-hTBP-I), as a soluble form of TNF receptor type I, is also reported to play a role in inhibiting the progression of endometriosis clinically (54, 55).

#### 9.2.2 Interleukin preparation

Interleukins, especially IL-12, are suggested to play an important role in the pathogenesis of endometriosis. IL-12 is reported to induce the production of other cytokines, promote the aggregation of inflammatory cells, and enhance NK cytotoxicity (56). In the mouse model of endometriosis, injection of IL-12 successfully inhibited the development of endometriosis lesions and enhanced the cytotoxicity of NK cells (57). In another study using the mouse endometriosis model, NK cells were found activated, and the formation of endometriosis lesions was found to be decreased after an intraperitoneal injection of IL-12 (58). IL-37 has been proven to inhibit the development of endometrial lesions in mice. This effect can be achieved through two ways. One is that rhIL-37 inhibits the expression of inflammatory factor IL-4 in mouse DCs, thereby increasing the proportion of Th1/Th2 cells and reducing the inflammatory reaction. The second is that rhIL-37 can induce the maturation of DCs by inhibiting the phosphorylation of STAT3, enhance the antigen-presenting ability of DCs, and facilitate other immune cells to play an immune role. Injecting rhIL-37 is regarded as a feasible method to inhibit the development of endometriosis theoretically by some authors (59).

#### 9.2.3 Anti-TGF-β preparation

The expression level of TGF-β1 in endometriosis tissues, peritoneal fluid, and the endometrial peritoneum was found to be significantly increased compared with the controls (60). The increase of TGF-β1 ligand level is suggested to be related to the decrease of immune cell activity in the peritoneum and the increase of survival, attachment, and invasion of ectopic endometrial cells. Thus, theoretically speaking, TGF-β1 is suggested to play a key role in the development of peritoneal endometriosis, and targeting this pathway may have therapeutic potential.

#### 9.2.4 The nuclear factor kappaB-targeted inhibitor

Nuclear factor kappaB (NF-κB) is a widely expressed transcription factor, which plays an important role in innate immunity and other processes involving cell survival, proliferation, and differentiation. NF-κB plays an important role in regulating the proinflammatory response of endometrial stromal cells in patients with endometriosis (61). Therefore, theoretically speaking, NF-κB is an excellent potential candidate for targeting the inflammatory response of ectopic endometriosis cells.

### 9.3 Other immunomodulators

#### 9.3.1 Interferon

Interferon is a protein produced to resist the invasion and replication of pathogens and is roughly divided into two categories: Type I interferon such as INF α/INF β and Type II interferon such as INF γ produced by T cells (62, 63). Type I interferon has been studied in endometriosis for a long time. This has been found to reduce the proliferation of fibroblasts and various cancer cell lines *in vitro*. Ingelmo et al. showed that intraperitoneal or subcutaneous injection of recombinant INF-2β significantly reduces the growth area of intraperitoneal endometriosis implants in rats (64).

#### 9.3.2 Danazol

In addition to playing a role in hormone regulation, danazol is also used as an immunosuppressant in the treatment of endometriosis (65). Danazol has been found to inhibit the proliferation of peripheral blood lymphocytes in ectopic endometriosis cell cultures activated by T-cell mitogen, but does not affect the macrophage-dependent T lymphocyte activation of B lymphocytes (66). Furthermore, danazol inhibits the production of IL-1 and TNF by monocytes in a dose-dependent manner (67) and inhibits the cytotoxicity of macrophage/monocyte-mediated susceptible target cells in patients with mild endometriosis (68).

#### 9.3.3 Complement system inhibitors

The complement system is an effective component of the innate immune system, which plays a very important role in the body’s defense, including the identification and elimination of pathogens. It has been found that C3 inhibitors are probably a novel promising candidate used for endometriosis treatment theoretically in two pathways: One, blocking the initial inflammatory signal cascade and, two, relieving the inhibition of C3 on NK cytotoxicity (69). In addition, the high level of C1q in the peritoneal fluid of endometriosis patients was reported to induce macrophages to differentiate into the MII phenotype, thus promoting angiogenesis in endometriosis injury. Therefore, specific blocking of C1q seems to play a role in enhancing macrophage activity (70).

#### 9.3.4 COX-2 inhibitor

Through regulation of hormones and hypoxia, the elevation of COX-2 levels in epithelial cells and stromal cells of ectopic endometriosis lesions leads to increased cell proliferation, invasion, angiogenesis, decreased apoptosis, and cytotoxic NK cell differentiation damage, thereby further promoting the pathogenesis and development of endometriosis (71). Therefore, COX-2 is suggested to be an important therapeutic target of anti-inflammatory drugs. COX-2 inhibitor treatment was documented to significantly reduce the microvessel density and the number of macrophages in endometriosis lesions and is related to the decreased expression of VEGF and Flk-1. The existing COX-2 inhibitors include naproxen and diclofenac, as well as new COX-2 selective inhibitors, such as celebrex, glycyrrhizin, and puerarin (72, 73). However, there have been no data on the clinical application of these agents in the treatment of endometriosis until now.

#### 9.3.5 Statins

Statins have been proven beneficial in the treatment of stromal cell growth in endometriosis lesions because of their inhibitory effects on the expression of some inflammatory genes (74). Previous studies have documented that atorvastatin significantly reduces the expression of inflammatory genes in endometriosis and reinforces the anti-inflammatory, anti-angiogenesis, and antioxidant properties of such drugs (75, 76). Monocyte chemoattractant protein 1 (MCP-1) is a chemokine that attracts and activates monocytes/macrophages. The data from an animal study pointed out that statins have an impact on the expression of MCP-1 in rat endometriosis implants (77). This result suggested that statins is probably a candidate in the treatment of endometriosis.

### 9.4 Mesenchymal stem cells

Mesenchymal stem cells (MSCs) secrete a wide range of bioactive molecules. Some of them are suggested to be responsible for regulating the immune inflammatory response and promoting lymphocyte differentiation, while others are observed to promote the regeneration and remodeling of damaged tissues (78). Published studies showed that IL-1β, IL-10, TNF, and IL-1R expression levels in the horse endometriosis model are generally decreased after receiving MSC treatment; however, IL-6 and IL-8 expression levels are increased, indicating that MSCs have both positive and negative regulatory effects on the inflammatory factors involved in the development of endometritis (79). The reduction of IL-1β and TNF is conducive to the downregulation of chronic inflammatory processes, which is helpful in the treatment of endometriosis (41, 80). This is likely the underlying immune inflammatory mechanism of how MSCs are involved in the treatment of endometriosis.

### 9.5 Vitamin D

Vitamin D has been documented to have immunomodulatory effects on the medical treatment of endometriosis (81). A recent experimental study on an animal model of endometriosis showed that the development of endometriosis and peritonitis is significantly inhibited after taking synthetic derivatives of vitamin D (82). Another study on the effect of vitamin D3 on endometrial implants in a rat model showed that taking vitamin D3 successfully reduced the cross-sectional area of endometriotic cysts by 48.8% (83). In view of the involvement of inflammatory cytokines in the pathogenesis of endometriosis, relevant studies have pointed out that the effect of 1,25(OH)2D3 on the production of cytokines in human endometrial stromal cells may be responsible for the therapeutic effect of this agent on endometriosis. Another research indicated that 1,25(OH)2D inhibits the Th1 cell response and promotes Th2 cell immune response by inhibiting the secretion of IL-12, IL-2, and TNF by T cells, macrophages, and DCs, respectively (84). These immune regulatory effects appeared to be effective in the medical treatment of endometriosis.

### 9.6 Bacillus Calmette–Guerin

The Bacillus Calmette–Guerin (BCG) vaccination induces xenoprotection against infection, cancer, and autoimmunity, not just *Mycobacterium* immunity (85). Research thus far has indicated that BCG vaccination reduces the number of implants in animal models of endometriosis, inhibits the implantation of endometriosis, and enhances peritoneal immune surveillance (57). BCG was also reported to activate cytotoxic NK cells, promote MI polarization to increase the proportion of MI/MII macrophages, and promote MII macrophages to secrete less IL-10 (86, 87). Data from the above studies provide us promising methods for the treatment of endometriosis.

## 10 Conclusion

Endometriosis is a common benign disease that mainly affects women of childbearing age. Despite being a common condition, its pathogenesis remains unclear. Many women will experience the phenomenon such as retrograde menstruation after menarche, but the incidence rate of endometriosis is low, which indicates that not all endometrial tissues can invade the pelvic and abdominal cavities. Moreover, it has been seen that the abnormal immune response mediated by immune cells causes ectopic growth of endometrial cells. Traditionally, medical treatment of endometriosis primarily includes surgery and hormone therapy. However, these methods are not satisfactory to date. The recurrence rate of this disease is high. Accumulating evidence suggests that several immunologic factors are probably involved in the pathogenesis of endometriosis. These factors directly or indirectly promote the development of endometriosis. These findings have provided novel insights into the treatment strategy of endometriosis. An increasing number of studies have suggested that the medical methods targeting these immunologic factors inhibit the development of endometriosis across different aspects (**Table 1**). This is probably a milestone in the treatment of this disease. However, information on the clinical application of immunotherapy in endometriosis is very limited and detailed mechanisms need to be further investigated. In the future, large-scale and multicenter clinical studies about immunotherapy in endometriosis should be considered. Furthermore, experimental studies about the mechanism of the immunotherapy applied in endometriosis are also needed.

**Table 1:** Summary of immunotherapy methods.

Classification	Therapeutic method	References
Mechanism of action	1. Anti-TGF-β preparation 2. The nuclear factor kappaB-targeted inhibitor 3. Complement system inhibitors	(60) (61) (69) (70)
Preclinical evidence	1. Immunotherapy based on the CD47SIRPa signaling pathway 2. Anti-exocrine therapy 3. Oral *Lactobacillus gasseri* OLL2809 4. An extract of the white mistletoe tree Helixor A 5. Targeted inhibition of PD-1 6. Loratadine 7. Anti-TNF preparation 8. Interleukin preparation 9. Recombinant INF-2β 10. COX-2 inhibitor 11. Statins 12. Mesenchymal stem cells 13. Vitamin D 14. Bacillus Calmette–Guerin	(39) (40) (42) (43) (47) (52) (53) (54) (55) (57) (58) (59) (64) (72) (73) (75) (76) (77) (79) (82) (83) (57)
Clinical evidence	1. Levonorgestrel-releasing intrauterine system 2. Danazol	(46) (68)

## Author contributions

AL and NM designed and arranged the manuscript. AL, WL and LQ wrote the article. XL, SY and JX identified and analyzed the references in Medline, participated in writing the paper, and provided final approval of the version to be published. NM revised the paper. All authors contributed to the article and approved the submitted version.
